# Constitutive expression of VviNAC17 transcription factor significantly induces the synthesis of flavonoids and other phenolics in transgenic grape berry cells

**DOI:** 10.3389/fpls.2022.964621

**Published:** 2022-07-29

**Authors:** Hélder Badim, Mariana Vale, Marco Coelho, Antonio Granell, Hernâni Gerós, Artur Conde

**Affiliations:** ^1^Department of Biology, Centre of Molecular and Environmental Biology, University of Minho, Braga, Portugal; ^2^Institute of Molecular and Cellular Biology of Plants, Spanish National Research Council (CSIC), Polytechnic University of Valencia, Valencia, Spain; ^3^Department of Biological Engineering, Centre of Biological Engineering, University of Minho, Braga, Portugal

**Keywords:** VviNAC17, homologous expression, anthocyanins, flavonoids, phenolics, secondary metabolites, grape berry cells

## Abstract

VviNAC17 is a grapevine transcription factor activated by ABA. Because ABA has been proposed as the main signal modulating the secondary metabolism in grape berry skins, here we postulated VviNAC17 as a positive regulator of secondary metabolism in grape cells. To validate the hypothesis, *VviNAC17* was constitutively and stably overexpressed in grape berry suspension-cultured cells of Gamay Fréaux cv. by *Agrobacterium*-mediated transformation. Targeted transcriptional analyses by qPCR showed that several genes involved the phenylpropanoid (*VviPAL1*), stilbenoid (*VviSTS1*) and flavonoid pathways (*VviDFR*, *VviLAR1*, *VviANR*, *VviLDOX*, and *VviUFGT1*), as well as anthocyanin vacuolar transport and accumulation (*VviGST4* and *VvMATE1*) were significantly upregulated in VviNAC17-overexpressing transgenic cells, which translated in the stimulation of a number of enzymatic activities in those pathways. This was the case of phenylalanine ammonia lyase (PAL) and UDP-glucose:flavonoid 3-O-glucosyltransferase (UFGT) that were about 2-fold and 3.5-fold higher in VviNAC17-overexpressing cells than in control cells. VviNAC17-overexpressing cells accumulated significantly higher amounts of anthocyanins, proanthocyanidins, total flavonoids and total phenolics. These findings confirmed that *VviNAC17* is an important positive regulator of secondary metabolism in grapevine contributing to the accumulation of important berry quality-related secondary metabolites.

## Introduction

Grapevine (*Vitis vinifera* L.) is a fruit crop with enormous economic importance worldwide (Bouquet et al., 2006). Wine industry is fundamental from both an economic and social standpoint, so, increasing knowledge about the biochemical and physiological features underlying the quality of grape berries, as well as about strategies for its improvement, is always a constant demand. This is especially important in the context of the current climate changes, responsible for alterations in the viticultural map but also for increasing environmental constraints. These have increasingly been tentatively mitigated through practical short-term strategies such as exogenous application of protective compounds as the kaolin for instance (Conde et al., 2016, 2018; Garrido et al., 2019), but also through long-term strategies as plant breeding (Ollat et al., 2018).

Plant primary metabolism is an essential biochemical process in the regulation of plant nutrition, while secondary metabolism is strongly involved in plant defense processes, such as biotic and abiotic stress responses. Secondary metabolites are very important in the modulation of fruit quality characteristics and, consequently, in the quality of wine. In this regard, phenolics, including their subgroups as flavonoids (e.g., anthocyanins and proanthocyanidins), are crucial to obtaining a variety of wine attributes such as color/pigmentation, aroma, flavor, astringency, and texture (Teixeira et al., 2013). The initial step of phenolic compounds synthesis occurs *via* the general phenylpropanoid pathway (GPP) and is followed by a series of metabolic reactions. The products originating from GPP are used as substrates by three distinctive pathways and converted into stilbenes, flavonoids, and phenolic acids (Deng and Lu, 2017; Rienth et al., 2021). The primary substrate of GPP, phenylalanine, is converted to *p*-coumaroyl-CoA *via* PAL, cinnamic acid-4-hydroxylase (C4H) and *p*-coumaric:CoA ligase (4CL). The product of GPP, *p*-coumaroyl-CoA, is further used as a substrate by stilbene synthase (STS) and chalcone synthase (CHS) in the first steps of the stilbenoid and flavonoid pathway, respectively. In the grapevine genome 45 stilbene synthases genes (*STS*) are currently described (Vannozzi et al., 2012). The biosynthesis of stilbenes occurs under the control of VviMYB14 and VviMYB15, two R2R3 MYB TFs (Höll et al., 2013; Rienth et al., 2021). In a competitive manner with stilbene synthases, chalcone synthases (CHS) are responsible for the first step of the flavonoid synthesis pathway to produce flavanones, such as naringenin (Ferrer et al., 2008). Flavanones are converted by flavanone-3β-hydroxylase (F3H) into dihydroflavonols. This class of compounds is used to produce flavonol aglycones *via* flavonol synthase (FLS) and it is regulated by the light-induced transcription factor VviMYBF1 (Downey et al., 2003; Czemmel et al., 2009; Revilla et al., 2018). By the competitive action of dihydroflavonol-4-reductase (DFR), dihydroflavonols are reshuffled away from flavonol biosynthesis branch and transformed into leucoanthocyanidins, which might be either converted by leucoanthocyanidin dioxygenase (LDOX) or leucoanthocyanidin reductase (LAR) into anthocyanidins or flavan-3-ols, respectively (Bogs et al., 2005; Czemmel et al., 2012). In grapevine, the regulation of *VviLAR1* and *VviANR* occurs consistently with the expression of two transcription factors, VviMYBPA1 and VviMYBPA2 (Bogs et al., 2005; Terrier et al., 2009; Rienth et al., 2021). Since the anthocyanidins are highly unstable molecules, these are immediately transformed either into anthocyanins or epicatechin by the competitive action of UDP-glucose: flavonoid-3-O-glucosyltransferase (UFGT) and anthocyanidin reductase (ANR; Bogs et al., 2005; Walker et al., 2007; Czemmel et al., 2012). The two most important transcriptional regulators of berry anthocyanin biosynthesis are the TFs VviMYBA1 and VviMYBA2 (Kobayashi et al., 2004; Walker et al., 2007; Rienth et al., 2021). The produced anthocyanins are transported across the tonoplast by primary or secondary transporters and accumulated in the vacuole. ATP-binding cassette (ABC), as is the case of VviABCC1, is responsible for the translocation of glycosylated anthocyanins (Francisco et al., 2013), and multidrug and toxic extrusion (MATE) transporters, like *VviMATE1*, are associated with the translocation of anthocyanin-acylglucosides (Gomez et al., 2009). Furthermore, glutathione S-transferases (*VviGSTs*), such as *VviGST1* and *VviGST4,* have been implicated in the stabilization and transport of anthocyanins to the vacuole (Pérez-Díaz et al., 2016).

The secondary metabolism is strongly linked to abiotic stress, especially to abscisic acid (ABA)-mediated responses (Ferrandino and Lovisolo, 2014). The mechanisms involving the abscisic acid perception and signaling in grapevine have been continuously studied over the last decade, leading to the identification of several associated molecular mechanisms such as ABA receptors, PP2Cs and SnRK2 kinases, NAC transcription factors (TFs) and AREB/ABF (ABF1, AREB1/ABF2, ABF3, AREB2/ABF4; Kang et al., 2002; Gambetta et al., 2010; Boneh et al., 2012a,b; Yoshida et al., 2015; Pilati et al., 2017; Ju et al., 2020).

NAC proteins are plant-specific transcriptional regulators implicated in the development of shoot apical meristems, floral organs and lateral shoots, as well as, in a very important manner, in the plant hormonal regulation, including ABA signaling and transduction and ABA-related defensive mechanisms (Ernst et al., 2004). These proteins include the NAC domain, an N-terminal module of *ca.* 160 amino acids, that is accompanied by diverse C-terminal transcriptional activation domains (Aida et al., 1997). The interaction between NAC regulatory proteins and hormone signaling is very complex, with numerous studies describing interactions with abscisic acid, jasmonic acid, salicylic acid, and ethylene-stress responses (Sun et al., 2013; Sakuraba et al., 2020; Figueroa et al., 2021). Therefore, NAC TFs are key intermediates responsible for the regulation of many downstream ABA-regulated targets. In grapevine, *VviNAC17* promoter is induced by ABA *via VviABF2* and, as *VviABF2*, and this TF, using transgenic *Arabidopsis* as a model, was shown to play as an activator of several molecular mechanisms regulated by ABA, as those involved in abiotic-stress tolerance responses (Pilati et al., 2017; Ju et al., 2020).

As ABA signaling is directly linked to the stimulation of several secondary metabolic pathways, in this study we hypothesized that VviNAC17 TF is a positive regulator of secondary metabolism in grapevine, stimulating the phenylpropanoid, flavonoid and stilbenoid pathways. To test this hypothesis, *VviNAC17* was constitutively and stably overexpressed in grape berry suspension-cultured cells of Gamay Fréaux cv., as a homologous grape model, through *Agrobacterium*-mediated transformation, and its function in the overproduction of target metabolites in those pathways and in the regulatory mechanisms was assessed.

## Materials and methods

### Plant material

Grape berry cells of *V. vinifera* L. cv. Gamay Fréaux cv. Teinturier, previously obtained and established from dedifferentiation of grape berry mesocarp and kindly provided by Prof. Serge Delrot (ISVV, Université de Bordeaux), were maintained in both solid and liquid cultures containing 0.1% (w/v) Gamborg B5 medium, 3% (w/v) sucrose, 250 mg L^−1^ casein hydrolysate, 0.2 mg L^−1^ kinetin and 0.1 mg L^−1^ naphthaleneacetic acid (NAA), in 250 ml flasks with shaking at 100 rpm at 23°C, under a 8 h dark/ 16 h light (200 μmol photons m^−2^ s^−1^) photoperiod. Cell suspensions were maintained by sub-culturing every 7 days by the transfer of 10 ml of cultures into 40 ml of fresh culture medium. As solid culture, *calli* were routinely sub-cultured every month.

### *VviNAC17* cloning and construction of destination vector

The open reading frame (ORF) of *VviNAC17* was cloned from grape berries of cv. Touriga Nacional. Its sequence was constituted by 1,002 bp and encoded 334 amino acids according to annotated transcripts of GSVIVG01014403001 (https://www.genoscope.cns.fr/externe/GenomeBrowser/Vitis/). *VviNAC17* was cloned using Gateway^®^ technology (Invitrogen). Primers with the attB sequences (Supplementary Table 1) for site-specific recombination with the entry plasmid *pDONR221* were utilized for PCR amplification. The stop codon was removed in the cloning process for allowing the subsequent *VviNAC17* C-terminus fusion with *egfp* in the destination vector, for later confirmation of successful transformation and constitutive expression, as well as confirmation of subcellular localization studies. Subsequently, recombination of *VviNAC17* containing attB sites with the entry plasmid was performed using BP clonase. *VviNAC17* in the entry plasmid was then recombined into the *pH7FWG2* using LR clonase. The correct assembly of the C-terminally fused to GFP construct *pH7FWG2:VviNAC17* was confirmed by sequencing.

### Stable transformation of Gamay cells with *pH7FWG2-VviNAC17* construct

*Agrobacterium* strain GV3101 was made chemically competent and transformed with the binary construct *pH7FWG2*-*VviNAC17* (Höfgen and Willmitzer, 1988). Bacterial cell culture of *Agrobacterium tumefaciens* containing the construct *pH7FWG2-VviNAC17* was cultivated on LB solid medium at 30°C for 48 h. A single colony was inoculated in 50 ml of liquid LB medium that contained 25 mg L^−1^ rifampicin and 100 mg L^−1^ spectinomycin and was incubated overnight at 28°C with shaking (180 rpm). Fifty ml of LB medium, supplemented with the same antibiotics, was inoculated with 1 ml of this 1-day-old culture and grown at 30°C with shaking (with shaking at 180 rpm) until OD_600_ reached around ≈ 0.1–0.4 (Genesys^™^ 20, Thermo Scientific^™^), as described in the literature (Wise et al., 2006). The bacterial medium was removed by centrifugation at 5,000 rpm for 5 min and the resulting bacterial pellet was washed and suspended in 1 ml of liquid Gamborg B5 medium. The transformation of Gamay cells was performed according to Martínez-Márquez et al. (2015) with some modifications. A 7-day-old Gamay cell suspension-cultured in Gamborg B5 medium was used to promote an efficient infection ratio between *A. tumefaciens* and Gamay cells healthy cells.

The liquid plant cell preparation was supplemented with 100 mg L^−1^acetosyringone. Subsequently, the bacterial suspension was added, and the infected culture was incubated on a shaker (100 rpm) for 30 min in the dark at 24°C. The suspension was then filtered, and the retained biomass washed with cold Gamborg B5 medium. Gentle vacuum was applied to remove excess of medium. The biomass was transferred to solid Gamborg B5 medium containing 100 mg L^−1^acetosyringone. After 2 days of co-culture incubation in the dark at 25°C, cells were transferred to solid Gamborg B5 medium containing 250 mg L^−1^cefotaxime and 5 mg L^−1^of hygromycin and kept at 25°C in the dark. Routine subculturing of the growing *callus* colonies was performed using decreasing cefotaxime concentrations. After 6 months, cefotaxime was eliminated from the medium and the transformed Gamay cells were grown in 8 mg L^−1^ of hygromycin.

### Subcellular localization

To analyze the subcellular localization of the VviNAC17 protein, transformed Gamay cells with the construct *pH7FWG2-VviNAC17-EGFP* were observed using fluorescence microscopy. Green fluorescence signals were observed and registered using the fluorescence microscope Leica BM5000B with an excitation wavelength of 488 nm and emission wavelength of 507 nm.

### Quantification of anthocyanins

The extraction of anthocyanins was performed using 100 mg of grape berry cells from each experimental condition as described in Vale et al. (2021). The extraction process was performed using 90% (v/v) methanol and 10% (v/v) deionized water. The suspensions were vigorously vortexed for 10 min and left overnight at 4°C for improved extraction. The samples were centrifuged at 18000 × *g* for 20 min. The supernatants were recovered and 200 μl of each extract were mixed with 1.8 ml of 25 mM KCl (pH = 1.0). The absorbance of samples for each experimental condition was measured at 520 and 700 nm with a Shimadzu UV-160A spectrophotometer (Kyoto, Japan). Total anthocyanin concentration was assessed as cyanidin-3-glucoside (C-3-G) equivalents, as described in the following equation:


$$\left[\mathrm{Total anthocyanins}\right]\left(\mathrm{mg}\;L^{-1}\right)=\frac{\left(A_{520}-A_{700}\right)\times \mathrm{MW}\times \mathrm{DF}\times 1000}{\varepsilon \times 1}$$

where MW is the molecular weight of C-3-G (449.2 g mol^−1^), DF corresponds to the dilution factor and ε is the molar extinction coefficient of C-3-G (26 900 M^−1^ cm^−1^). The concentration was subsequently presented per mg of dry weight (DW) of used cells.

### Quantification of total flavonoids

Quantification of flavonoids was performed using the aluminum chloride colorimetric method described by Matejić et al. (2013) with some modifications. An assay mixture containing 20 μl of standard solutions (quercetin) or methanolic extracts from the experimental conditions, 30% (v/v) of ethanol, 0.2% (w/v) of aluminum chloride and 20 mM of potassium acetate was used. Following incubation at room temperature during 40 min in the dark, the absorbance of the reaction mixtures was measured at 415 nm with a Shimadzu UV-160A spectrophotometer (Kyoto, Japan). Total flavonoid concentration in methanolic extracts was calculated using a quercetin calibration curve (5–100 μg mL^−1^) and expressed as quercetin equivalents per gram of DW.

### Quantification of proanthocyanidins

Proanthocyanidins (PAs) concentration was quantified resorting to an adapted colorimetric DMAC assay based on Wallace and Giusti (2010). For extraction, 1 ml of absolute methanol was added to 5 mg of lyophilized grape berry cells and vigorously shaken for 30 min followed by centrifugation at 18000 × *g* for 30 min. DMAC reagent was prepared immediately before use by dissolving 2% DMAC (w/v) in a 1:1 ratio of 6 N H_2_SO_4_ and methanol absolute solution. The reaction mixture containing 1.175 ml of methanol and 50 μl of 2% (w/v) DMAC was initiated by adding 10 μl of extract of each experimental condition. Each reaction was incubated at room temperature during 15 min in the dark and the absorbance was determined at 640 nm using a Shimadzu UV-160A spectrophotometer (Kyoto, Japan). Total proanthocyanidins concentration was determined using a calibration curve with (+)-catechin standard solutions (50–500 μg mL^−1^) and expressed as catechin equivalents per gram of DW.

### Quantification of total phenolics

The concentration of phenolics was quantified by Folin–Ciocalteu colorimetric method as previously performed in Conde et al. (2016). Total phenolics were extracted in 1.5 ml of 90% (v/v) methanol from 20 mg of lyophilized control and transformed grape berry cells. The homogenates were vigorously shaken for 30 min and left overnight at 4°C. Subsequently, each experimental condition was centrifuged at 18000 × *g* for 20 min. Twenty μl of each supernatant were mixed with 1.58 ml of deionized water and 100 μl of Folin reagent, shaken and incubated in the dark for 5 min before the addition of 300 μl of 2 M sodium carbonate. After 2 h of incubation in the dark, the absorbance was quantified at 765 nm (Shimadzu UV-160A spectrophotometer, Kyoto, Japan). Total phenolic concentrations were determined as gallic acid equivalents (GAE) per gram of dry weight.

### RNA extraction from Gamay cells and cDNA synthesis

Approximately 200 mg of fresh control and VviNAC17-overexpressing Gamay cells were used for total RNA extraction, *via* the method described by Reid et al. (2006) in combination with the GRS Plant Total RNA kit (GRISP). The extraction buffer was modified to 2% (w/v) cethyl-trimethylammonium bromide (CTAB), 2% (w/v) soluble polyvinylpyrrolidone (PVP) K-30, 300 mM Tris–HCl (pH 8.0), 25 mM EDTA, 2 M NaCl and 40 mM dithiothreitol (DTT). Following an in-column DNase I (Thermo Scientific^™^) treatment, the RNA integrity was confirmed in a 1% (w/v) agarose gel. Complementary DNA (cDNA) was synthesized from 1 μg of total RNA using the Xpert cDNA Synthesis Master-mix Kit (GRISP) according to the manufacturer’s instructions. RNA purity and concentration were assessed using NanoDrop.

### Transcription analyses by real-time qPCR

Quantitative Real-time PCR analyses (qPCR) were performed using Xpert Fast SYBR Blue (GRISP) and a CFX96 Real-Time Detection System (Bio-Rad) using 1 μl of cDNA in a reaction volume of 10 μl per well. Experiments were carried out in biological triplicates using *VviACT1*(actin) and *VviGAPDH* (glyceraldehyde-3-phosphate dehydrogenase) as reference genes, verified as stable and ideal for qPCR normalization in grapevine (Reid et al., 2006). Melting curves were performed after each run to confirm the absence of unspecific and primer-dimer amplification. Gene expression values were normalized by the average of expression of the reference genes, as described by Pfaffl (2001), and analyzed using the CFX Manager Software 2.0 (Bio-Rad). Specific primer pairs used in this work are listed in Supplementary Table 2.

### Protein extraction

Total protein extraction from grape berry cells was performed as in Conde et al. (2016). Sample powder was vigorously mixed with extraction buffer at a1:2 (v/v) powder: buffer ratio. Protein extraction buffer contained 50 mm Tris–HCl pH 8.9, 5 mm MgCl_2_, 1 mm EDTA, 1 mm phenylmethylsulfonyl fluoride (PMSF), 5 mm dithiothreitol (DTT), 0.1% (v/v) Triton X-100 and 1% of poly(vinylpolypyrrolidone; PVPP). Homogenates were vigorously mixed for 20 min and centrifuged at 18000 × *g* for 20 min at 4°C. The supernatants were kept on ice and used for all enzymatic assays. Protein concentration of the crude extracts was quantified by the Bradford method (Bradford, 1976), with bovine serum albumin as standard.

### UDP-glucose:flavonoid 3-O-glucosyltransferase and phenylalanine ammonia lyase enzyme activity assays

Maximum velocity (*V*_max_) of UDP-glucose:flavonoid 3-O-glucosyltransferase (UFGT) activity was determined as in Lister et al. (1996) and adapted by Conde et al. (2016). The assay mixture contained 300 mm Tris–HCl buffer pH 8.0 (optimal activity), 1 mM UDP glucose and 200 μl of enzyme extract, and 5 mm of DTT in a reaction volume of 1 ml. The reaction was started with 1 mm quercetin as substrate, thus at a saturating concentration. The reaction mixture was kept in the dark under gentle shaking for 30 min. Following incubation, dilutions were prepared with 100 μl of each assay mixture and 900 μl of Tris–HCl buffer and the production of quercetin-3-glucoside was assessed at 350 nm (*ε* = 21,877 M^−1^ cm^−1^) with a Shimadzu UV-160A spectrophotometer (Kyoto, Japan).

Maximum velocity (*V*_max_) of phenylalanine ammonia lyase (PAL) activity was measured as in Conde et al. (2016). The assay mixture contained 0.2 ml of enzyme crude extract and 3.6 mm NaCl in 50 mm Tris–HCl pH 8.9 of reaction buffer in a reaction volume of 1 ml. Reactions were started by adding 25 mm of L-phenylalanine. The reaction mixture was kept at 41°C in the dark under gentle shaking for 30 min and the production of trans-cinnamic acid was assessed at 290 nm (*ε* = 17,400 M^−1^ cm^−1^).

### Statistical analysis

**Figure 1:**
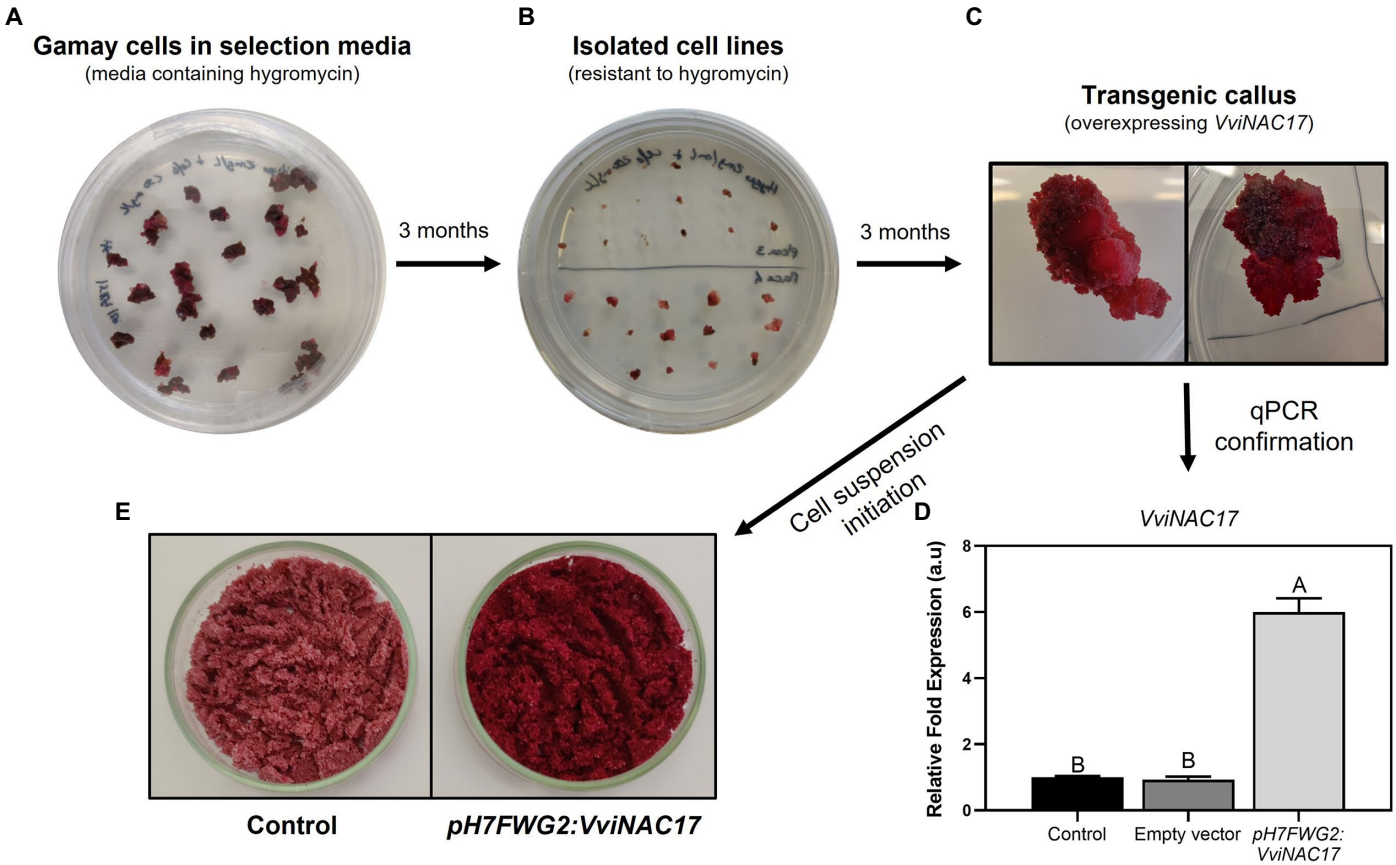
Transformation of non-embryogenic cell culture of *Vitis vinifera* Gamay overexpressing the transcription factor VviNAC17. **(A)** Transformed callus obtained after infection with *Agrobacterium tumefaciens* in selection media containing hygromycin and cefotaxime. **(B)** Multiple isolated cell lines obtained after 3 months of growth in selection media containing hygromycin and cefotaxime. **(C)** Transgenic callus after 3 months of growth. **(D)** Real-time qPCR demonstrating the upregulation of *VviNAC17* in *pH7FWG2-VviNAC17* Gamay cells. Statistical differences are denoted by different letters and were determined by one-way ANOVA and Tukey’s *post hoc* test. **(E)** Cell suspensions of control and *pH7FWG2*-*VviNAC17*-containing Gamay cells filtered after 7 days of growth in Gamborg B5 medium.

Except for the results pictured in Figure 1D, for which a one-way ANOVA test and Tukey’s *post-hoc* test were used, results were analyzed by Student’s *t-*test using GraphPad Prism 9 software (GraphPad Software Inc., United States). The results are presented as mean ± standard deviation of the mean (SD), and statistical comparisons were made with a 95% confidence interval. Parametric testing was used as all data sets have Gaussian distributions. Statistical significance is indicated in the figures according to value of *p*, with *p* < 0.05 (^*^), *p* < 0.01 (^**^), *p* < 0.001(***) or *p* < 0.0001(^****^). For every experimental approach, at least three biological replicates were used.

## Results

### Establishment of non-embryogenic grape cell cultures of Gamay cv. constitutively expressing the transcription factor VviNAC17

The transgenic grape cell line constitutively expressing VviNAC17, under the control of 35S cauliflower mosaic virus (CaMV) promoter, was obtained and established after transformation with the *A. tumefaciens* GV3101 strain, previously transformed with the construct *pH7FWG2:VviNAC17* (Figures 1A–C). Six months after transformation, Hygromycin-selected Gamay transgenic and non-transformed *calli* were checked for plant genome T-DNA integration of *VviNAC17* by PCR amplification using a specific primers pair for amplification of the promoter 35S (Supplementary Table 1), and its detection was confirmed to occur only in transgenic clones (Supplementary Figure 1). A transcriptional analysis by real-time qPCR confirmed the overexpression of *VviNAC17* in the transgenic callus transformed with *pH7FWG2:VviNAC17* (Figure 1D). The transgenic *callus* with the highest *VviNAC17* transcripts abundance was selected for further studies. Six months after the initial transformation and selection in media containing hygromycin and cefotaxime, sufficient transgenic *callus* was obtained to initiate a suspension culture (Figure 1E).

Besides evident phenotypic changes marked by a deep and more intense purple color than control Gamay cells, the transgenic VviNAC17-overexpressing suspension culture interestingly exhibited a substantial increase in cell growth rate when compared to non-transformed cell lines when cultivated in Gamborg B5 medium (Supplementary Figure 2).

### Subcellular localization of transcription factor *VviNAC17* in transgenic Gamay cells

**Figure 2:**
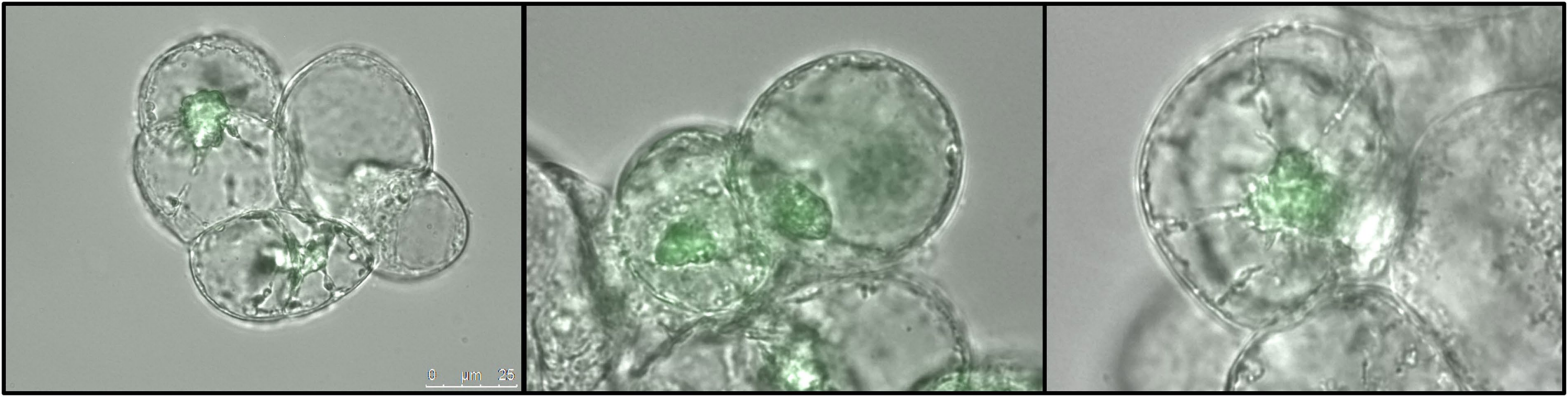
Subcellular localization of transcription factor VviNAC17 in *V. vinifera* Gamay transgenic cells. Distribution of the transcription factor VviNAC17 through the nucleus of transgenic VviNAC17-overexpressing Gamay cells 6 months after transformation with *A. tumefaciens* with the binary vector *pH7FWG2-VviNAC17*. Green fluorescence observed by fluorescence microscopy indicated the presence and expression of VviNAC17-GFP fusion protein in the nucleus and further confirmed the successful transformation of the cells. Bar of photographs—25 μm.

To further confirm VviNAC17-GFP recombinant protein synthesis in the transgenic cultures, a small fraction of tissue from transgenic *calli* was observed in a fluorescence microscope. As shown in Figure 2, green fluorescence was clearly observed in the nucleus of transgenic VviNAC17-overexpressing Gamay cells confirming the constitutive expression of VviNAC17 in transgenic cells, and also indicating its subcellular localization in the nucleus as expected. The fluorescence signal was absent in non-transformed cells.

### The effect of the overexpression of *VviNAC17* on the phenylpropanoid, stilbenoid and flavonoid pathways

**Figure 3:**
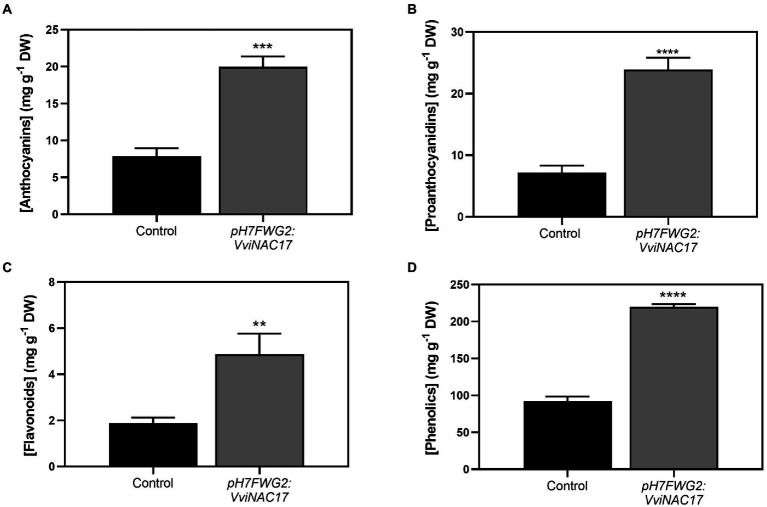
Effect of the overexpression of *VviNAC17* on total anthocyanin **(A)**, proanthocyanidins **(B)** flavonoid **(C)** and phenolics **(D)** content in transgenic cell suspensions of Gamay. Total anthocyanin concentration is represented by cyanidin-3-glucoside equivalents per g of dry weight (DW). Total proanthocyanidin concentration is represented by (+)-catechin per g of dry weight (DW). Total flavonoids concentration is represented by quercetin equivalents per g of dry weight (DW). Total phenolics concentration is represented by gallic acid equivalents (GAE) per g of dry weight (DW). Values are the mean ± SD of three biological replicates. Asterisks indicate statistical significance between non-transformed cells (control) and transformed Gamay cells (*pH7FWG2-VviNAC17*). (Student’s t-test; ^**^p ≤ 0.01, ^***^p ≤ 0,001 and ^****^p ≤ 0.0001).

These analyses revealed a significant increase in the concentration of these compounds in the transgenic VviNAC17-overexpressing cells. Indeed, the concentration of anthocyanins in *pH7FWG2-VviNAC17* berry cells was 19.96 mg of cyanidin-3-glucoside equivalents per gram of cells dry weight compared to 7.88 mg of cyanidin-3-gluoside equivalents per gram of dry weight (DW) in control cells, which corresponded approximately to a 2.5-fold increase (Figure 3A). The concentration of proanthocyanidins in control cells was 7.22 mg of (+)-catechin equivalents per gram of DW compared to 23.88 mg of (+)-catechin equivalents per gram of DW in VviNAC17-overexpressing cells (Figure 3B; 3.5-fold higher). VviNAC17-overexpressing cells also had a 2.5-fold higher concentration of flavonoids, reaching 4.88 mg of quercetin equivalents per gram of DW (Figure 3C). The concentration of total phenolics in transformed cells was also increased by approximately 2.5-fold, reaching 219.84 mg of gallic acid equivalents per gram of DW (Figure 3D).

**Figure 4:**
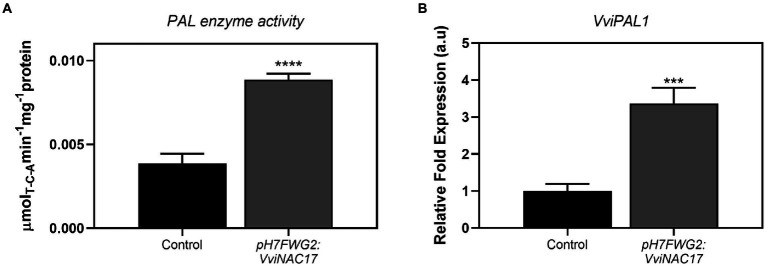
Stimulatory effect of the overexpression of *VviNAC17* in Gamay cells on the phenylpropanoid pathway. **(A)** Phenylalanine ammonia lyase (PAL) enzyme activity determined as *V_max_* in non-transformed (control) and *VviNAC17*-overexpressing Gamay cells (*pH7FWG2-VviNAC17*). Values are the mean ± SD of five biological replicates. Asterisks indicate statistical significance (Student’s t-test; ^****^p ≤ 0.0001). **(B)** Transcript abundance of phenylalanine ammonia lyase 1 (*VviPAL1*) in non-transformed (control) and *VviNAC17*-overexpressing Gamay cells (*pH7FWG2:VviNAC17*). Gene expression analysis was performed by real-time qPCR in non-transformed (control) and *VviNAC17*-overexpressing Gamay cells (*pH7FWG2:VviNAC17*). *VviPAL1* relative expression levels were obtained after normalization with the expression of the reference genes *VviACT1* and *VviGAPDH.* Values are the mean ± SD of three biological replicates. Asterisks indicate statistical significance (Student’s t-test; ^***^p ≤ 0.001, ^****^p ≤ 0.0001).

**Figure 5:**
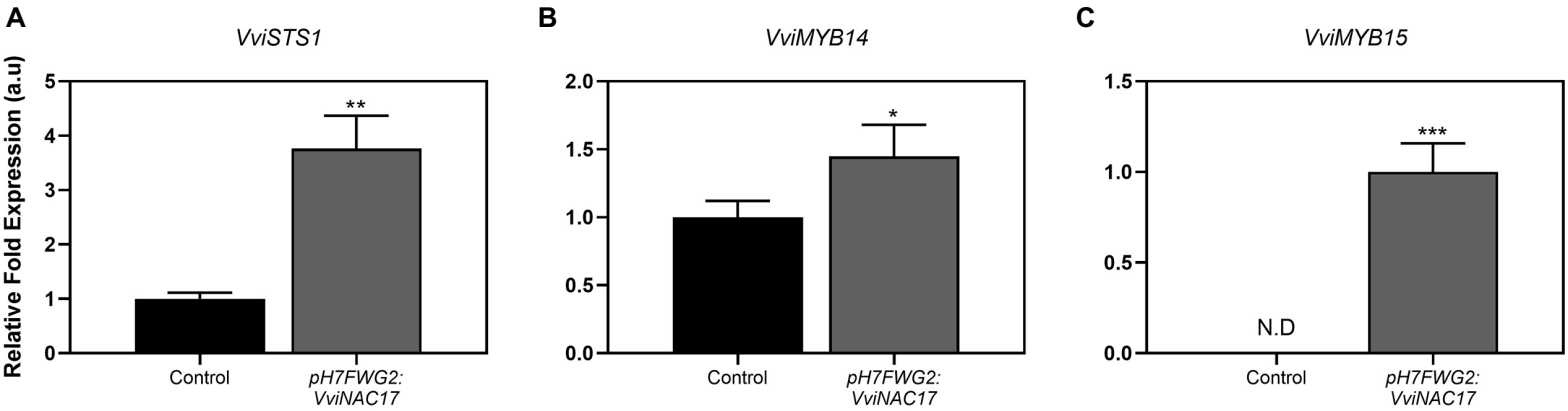
Effect of *VviNAC17* overexpression in the transcript levels of grapevine stilbene synthase 1 (*VviSTS1*) **(A)** and transcription factors *VviMYB14* **(B)** and *VviMYB15* **(C)**. Gene expression analysis was performed by real-time qPCR in non-transformed (control) and *VviNAC17*-overexpressing Gamay cells (*pH7FWG2:VviNAC17*). *VviSTS1*, *VviMYB14* and *VviMYB15* relative expression levels were obtained after normalization with the expression of the reference genes *VviACT1* and *VviGAPDH*. Values are the mean ± SD of three biological replicates. Asterisks indicate statistical significance (Student’s t-test; ^*^p ≤ 0.05, ^**^p ≤ 0.01, ^***^p ≤ 0.001).

*VviNAC17*-overexpressing Gamay cells exhibited a 2-fold higher PAL specific activity when compared to control cells (Figure 4A), reaching a *V*_max_ of 0.009 μmol min^−1^mg protein^−1^. In accordance with the biochemical activity of PAL, the expression of *phenylalanine ammonia lyase 1* (*VviPAL1*) gene was approximately 3.5-fold higher in transgenic Gamay cells than in control cells (Figure 4B). The transcription of *stilbene synthase 1* (*VviSTS1*), the most expressed STS gene in grapevine (Ciaffi et al., 2019), increased up to 4-fold in *VviNAC17*-overexpressing Gamay cells, in synchrony with the increase in the expression of the transcription factors *VviMYB14* and *VviMYB15*, both positive regulators of *VviSTS1* and other stilbene synthases in grapevine (Höll et al., 2013; Figure 5).

**Figure 6:**
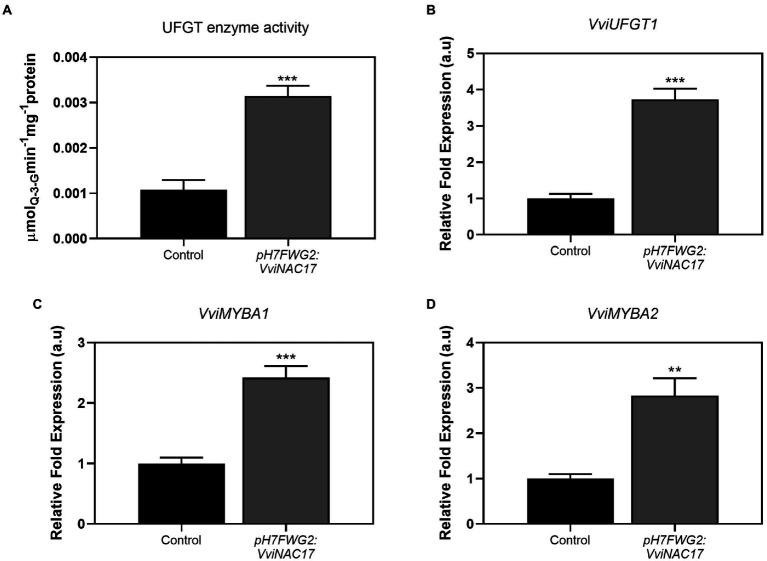
Stimulatory effect of the overexpression of *VviNAC17* on the synthesis of anthocyanins. **(A)** UDP-glucose:flavonol 3-O-glucosyltransferase (UFGT) total enzyme activity determined as *V_max_* in non-transformed (control) and *VviNAC17*-overexpressing Gamay cells (*pH7FWG2:VviNAC17*). Values are mean ± SD of the five biological replicates. Asterisks indicate statistical significance (Student’s t-test; ^***^p ≤ 0.001). **(B)** Transcript levels of UDP-glucose:flavonol 3-O-glucosyltransferase (*VviUFGT1*), *VviMYBA1* **(C)** and *VviMYBA2* **(D)** from R2R3 MYB family in non-transformed (control) and *VviNAC17*-overexpressing Gamay cells. *VviUFGT1*, *VviMYBA1* and *VviMYBA2* relative expression levels were obtained after normalization with the expression of the reference genes *VviACT1* and *VviGAPDH*. Values are presented as mean ± SD of three biological replicates. Asterisks indicate statistical significance (Student’s t-test; ^**^p ≤ 0.01, ^***^p ≤ 0.001).

The activity of UFGT enzyme was 3-fold higher in cells overexpressing *VviNAC17* when comparing with control cells, reaching a *V_max_* of 0.003 μmol min^−1^mg protein^−1^ (Figure 6A), in agreement with the significant increase in the total concentration of anthocyanins previously depicted in Figure 3A. In the same way, the expression of *VviUFGT1* was strongly stimulated in *VvNAC17*-overexpressing Gamay cells, reflected by a 4-fold increase in the transcripts abundance (Figure 6B). Two transcription factors associated with the positive regulation of *VviUFGT1*, *VviMYBA1* and *VviMYBA2*, also displayed a significantly higher expression in transformed cells, by 2.5-and 3-fold, respectively (Gollop et al., 2002; Figures 6C,D).

**Figure 7:**
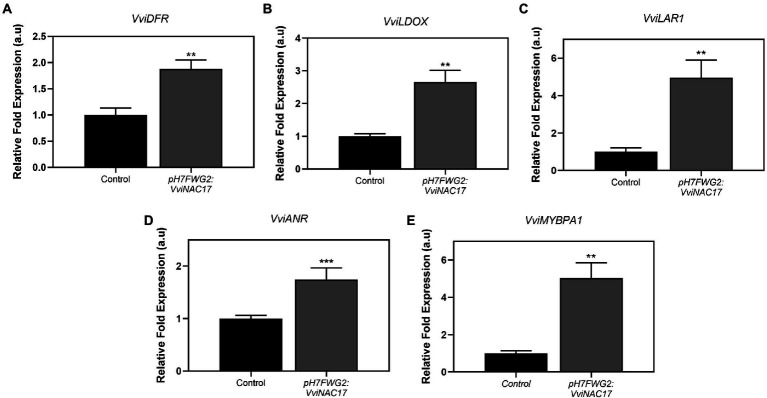
Stimulatory effect of the overexpression of VviNAC17 in the transcript levels of grapevine dihydroflavonol-4-reductase (*VviDFR*) **(A)**, leucoanthocyanidin dioxygenase (*VviLDOX*) **(B)**, leucoanthocyanidin reductase 1 (*VviLAR1*) **(C)**, anthocyanidin reductase (*VviANR*) **(D)** and *VviMYBPA1* **(E)**. *VviDFR, VviLDOX, VviLAR1, VviANR* and *VviMYBPA1* relative expression levels in non-transformed (control) and *VviNAC17*-overexpressing Gamay cells were obtained after normalization with the expression of the reference genes *VviACT1* and *VviGAPDH*. Values are presented as mean ± SD of three biological replicates. Asterisks indicate statistical significance (Student’s t-test; ^**^p ≤ 0.01).

A significant stimulation of *VviDFR* transcripts, corresponding a 2-fold increase, was observed in transformed Gamay cells when compared to the control (Figure 7A). The transcripts abundance of *VviLDOX* were also significantly higher in transformed cells compared to control cells, corresponding a 2.5-fold increment in cells overexpressing *VviNAC17* (Figure 7B). Specific targets associated with the synthesis of flavan-3-ols were also upregulated in transgenic cells, namely *VviLAR1* and *VviANR* as shown in Figures 7C,D. The expression of the gene encoding for the transcription factor VvMYBPA1, involved in the activation of LAR and ANR was also strongly upregulated in VviNAC17-overexpressing cells (Figure 7E).

### Transcriptional changes in anthocyanin S-conjugation and vacuolar transport

**Figure 8:**
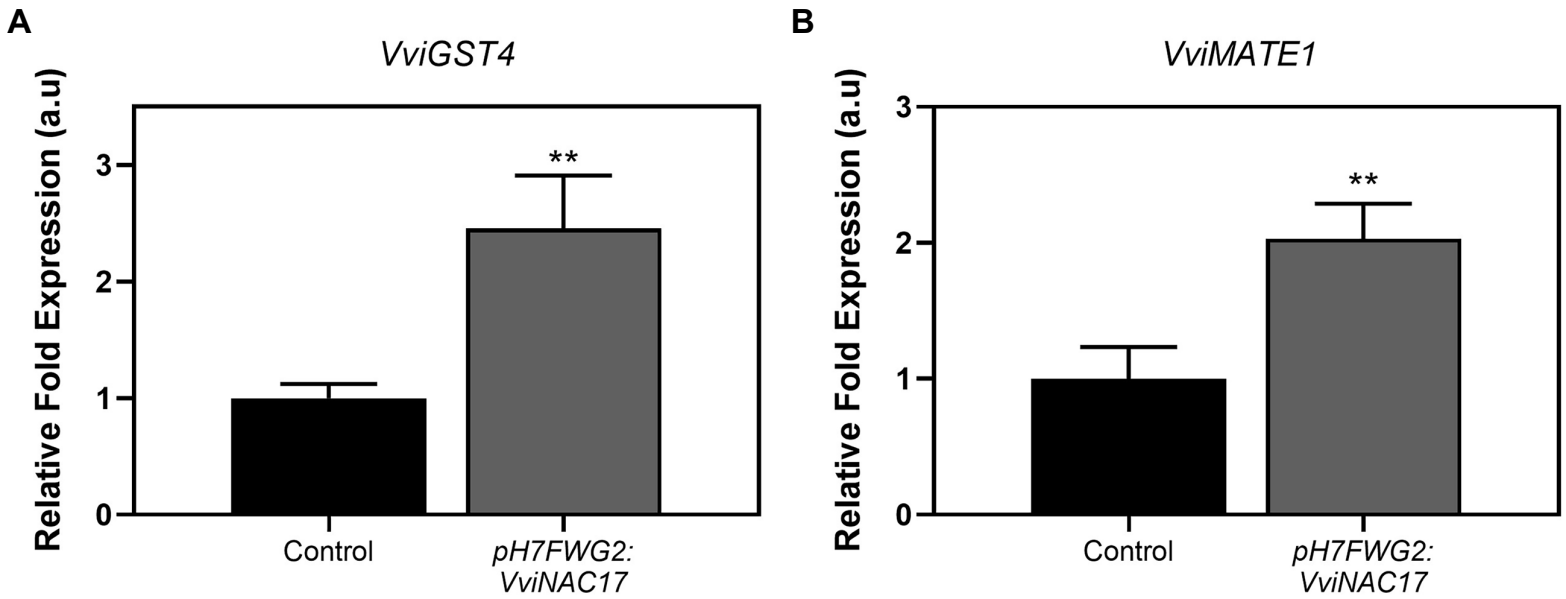
Stimulatory effect of the overexpression of VviNAC17 in the transcript levels of genes involved in anthocyanin S-conjugation (*VviGST4*) **(A)** and vacuolar transport capacity (*VviMATE1*) **(B)**. *VviGST4* and *VviMATE1* relative expression levels in non-transformed (control) and *VviNAC17*-overexpressing Gamay cells were obtained after normalization with the expression of the reference genes *VviACT1* and *VviGAPDH*. Values are presented as mean ± SD of three biological replicates. Asterisks indicate statistical significance (Student’s t-test; ^**^p ≤ 0.01).

The expression of *VviGST4* was significantly higher in transformed cells, with a 2.5-fold increase in transgenic cells compared to control cells (Figure 8A). This enzyme represents a crucial step in anthocyanin stabilization through the conjugation with a reduced form of glutathione (GSH), essential for most of the anthocyanin vacuolar transport (Conn et al., 2008). Gene expression of the tonoplast anthocyanin transporter *VviMATE1* was also highly upregulated in cells overexpressing *VviNAC17*, by 2-fold, compared to control cells (Figure 8B).

## Discussion

As mentioned in the Introduction section, using *Arabidopsis* as a model, *VviNAC17* was recently described as a stress-responsive gene regulated by ABA and implicated in resistance to abiotic stress, by activating several stress-responsive genes (Pilati et al., 2017; Ju et al., 2020). However, the role of VviNAC17 in the regulation of secondary metabolism, hence, several ABA-regulated grape berry quality-related molecular mechanisms was so far unknown. Here, using grape berry cells of Gamay Fréuax cv. as homologous model, by successfully constitutively and stably expressing VviNAC17, we demonstrated that this TF is a positive regulator of several secondary metabolism biochemical steps through the activation of key genes involved in phenylpropanoid and flavonoid pathways, using transcriptional and biochemical analysis. Indeed, the synthesis and concentration of some of the main classes of secondary metabolites synthesized in the above-mentioned biochemical pathways, namely, total phenolics, flavonoids and, among them, total anthocyanins and total proanthocyanidins, were significantly increased in Gamay grape berry cells constitutively overexpressing VviNAC17. In fact, VviNAC17-overexpressing Gamay cells exhibited an impressive concentration of phenolic compounds of over 200 mg g DW^−1^ in a clear demonstration of a very active secondary metabolism induced by VviNAC17. This concentration is significantly higher than, for instance, the 40 mg g DW^−1^ phenolics, previously observed in Gamay suspension-cultured cells (Noronha et al., 2020). Also remarkable was the fact that despite the notorious increase in the concentration of these secondary metabolites, VviNAC17-overexpressing cells displayed a higher growth rate than control cells. This demonstrates that the stimulation of secondary metabolism did not result from a tradeoff with a slower cell growth. While this is apparently surprising, it may be an indicator that the uptake of carbon/energy sources and/or primary metabolism was also enhanced by VviNAC17 overexpression, a hypothesis that needs to be further explored.

Among this extensive secondary metabolism upregulation, the stimulation of the phenylpropanoid pathway was notorious in *pH7FWG2:VviNAC17-*overexpressing cells as confirmed by the higher expression of *VviPAL1* compared to non-transformed cells, together with the higher PAL enzyme activity in transgenic cells, that is the result from the joint activity of all isozymes. Also, the upregulation of genes associated with the biosynthesis of stilbenes was evident in cells overexpressing *VviNAC17*, particularly the *VviSTS1*, and two TFs associated with the positive regulation of this particular pathway, *VviMYB14* and *VviMYB15* (Höll et al., 2013). While the upregulated expression of *VviSTS1* and of its activating TFs *VviMYB14* and *VviMYB15* is suggestive of a complete stimulation of the stilbenoid pathway in VviNAC17-overexpressing cells that translates into increased amounts of stilbenes, this cannot be unequivocally concluded, as the total concentration of stilbenes was not quantified in this study and transcriptional modifications are not always translated into metabolic changes.

As referred to above, the total amount of flavonoids was higher in VviNAC17-overexpressing cells. This group of compounds includes anthocyanins, a group of water-soluble flavonoid compounds produced by almost vascular plants, and known by a diverse range of biological functions (Rinaldo et al., 2015). In grapevine, anthocyanins are responsible for berry color, one of the most important quality traits of fruit and red wines production. The increased content of anthocyanins in VviNAC17-overexpressing cells can be explained by the higher UFGT enzyme activity and *VviUFGT1* expression levels in transgenic cells, a gene involved in anthocyanin biosynthesis by conversion of glycolates anthocyanidins into anthocyanins. Also, the initiation of UFGT expression is specifically regulated by two transcription factors, *VviMYBA1* and *VviMYBA2* (Walker et al., 2007). These two TFs were also upregulated in transgenic cells, which also corroborates with the higher expression levels of *VviUFGT1* and with the higher anthocyanin content in cells overexpressing *VviNAC17*. The regulatory mechanism between *VviNAC17* and MYB TFs is not well described, however several are the studies indicating a complex signaling network between members of NAC and MYB family (Ye et al., 2018; Chen et al., 2021).

The enzyme DFR is specific for the anthocyanin/proanthocyanidins pathway through the production of leucoanthocyanidins. This product is used as substrate by the enzyme LDOX, by converting leucoanthocyanidins to anthocyanidins. By transcriptional analysis, we demonstrated an upregulation of these two genes on transgenic cells, which supports the idea that the *VviNAC17* is intrinsically associated to the regulation of secondary metabolism, namely on those targets implicated in the synthesis of anthocyanins.

The third main class of flavonoids family, the proanthocyanidins (flavan-3-ols), have been demonstrated to be very active as defense molecules to protect plants against stress but also have beneficial health effects for humans (Dixon et al., 2005). Also, grapevine berry mesocarp and seed derived proanthocyanidins greatly affect the organoleptic properties of the red wine – associated with increased content, degree of polymerization and galloylation of proanthocyanidins (Vidal et al., 2003; Yu et al., 2019). In this study, transgenic cells overexpressing *VviNAC17* transcription factor exhibited upregulated levels of key genes implicated in proanthocyanidins biosynthesis, namely *VviLAR1*, *VviANR* and *VviMYBPA1*, but, even more importantly, a significantly increased concentration of PAs.

Anthocyanins are accumulated in the vacuole *via* primary transporters from the ABC family (Francisco et al., 2013) and tonoplast secondary transporters like *VviMATE1* of multidrug and toxic extrusion family (Gomez et al., 2009). Also, members of multigenic GST family have been described to be crucial for anthocyanin accumulation. In grapevine, only *VviGST1* and *VviGST4* are involved in anthocyanin accumulation in the vacuole (Pérez-Díaz et al., 2016). Namely, *VviGST4* is crucial for anthocyanin stabilization and transport, by promoting anthocyanin S-conjugation with reduced glutathione (Conn et al., 2008). In this study, we observed an increased expression of genes related with stabilization and transportation of anthocyanins to the vacuole, specifically, *VviMATE1* and *VviGST4*, respectively. Cutanda-Perez et al. (2009) demonstrated that the ectopic expression of *Vitis labrusca VlMYBA1-2* activates the gene expression linked to anthocyanin MATE transporter and GST. For this reason, the upregulation of *VviMYBA1* and *VviMYBA2* might also explain the increased levels of transcripts related with anthocyanin transport. On the other hand, the expression of *VviABCC1* was not affected by the overexpression of *VviNAC17* in transgenic cells.

**Figure 9:**
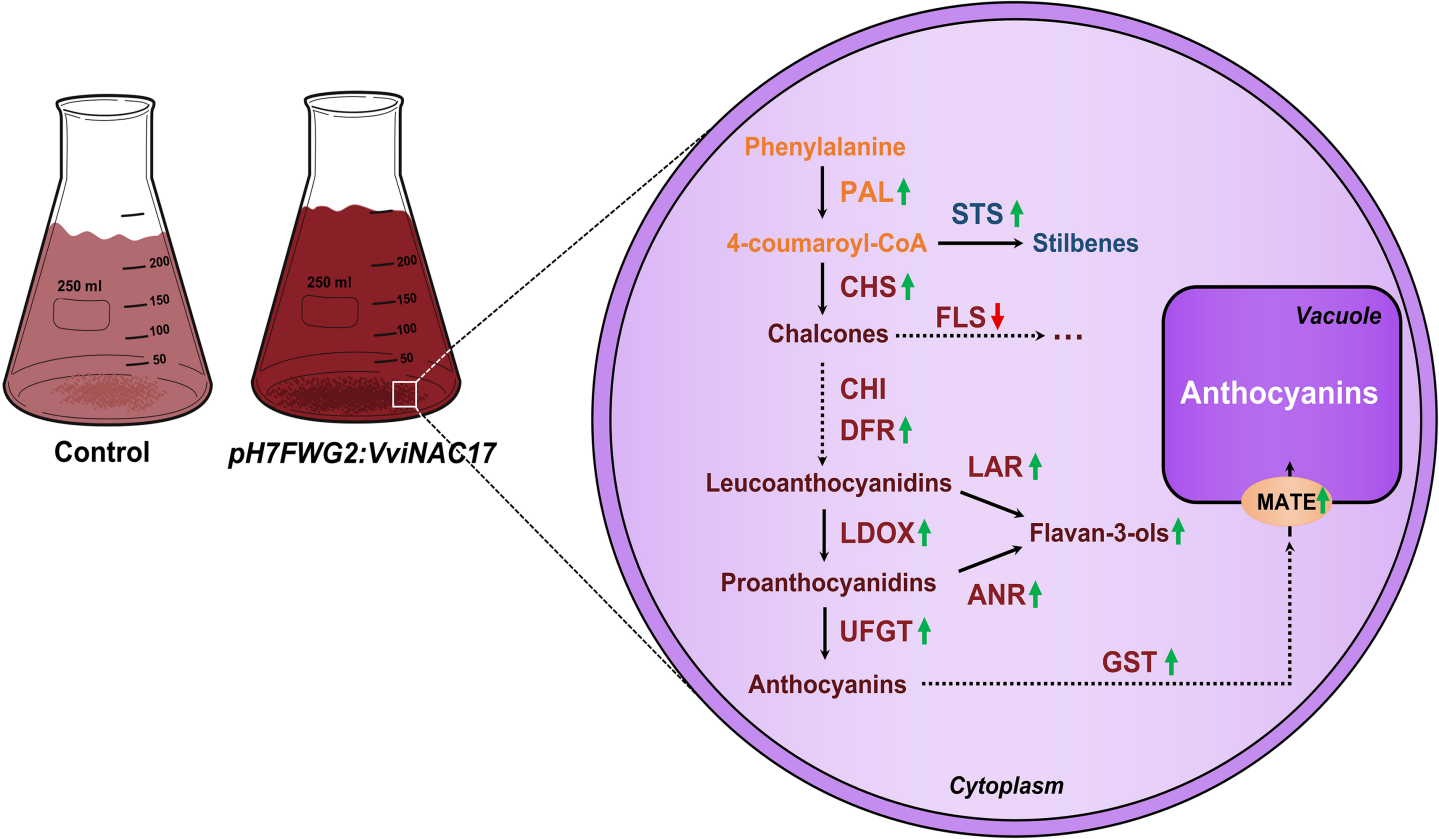
*VviNAC17* plays a role in stimulating the synthesis of anthocyanins and flavan-3-ols (PAs) in grape berry cells. The modulation of secondary metabolism, namely phenylpropanoid (PAL), stilbene (STS) and flavonoid (CHS, CHI, DFR, LAR, LDOX, ANR and UFGT) pathways, is positively regulated by the transcription factor VviNAC17. Also, genes involved in the vacuolar transport and accumulation of anthocyanins (GST and MATE) are positively regulated by *VviNAC17*.

In sum, we demonstrated that *VviNAC17* is a transcription factor associated with a broad positive regulation of secondary metabolism in grapevine, particularly on the pathways regulated by ABA. We observed that Gamay cells overexpressing *VviNAC17*, together with significantly higher concentrations of total phenolics, flavonoids, anthocyanins and proanthocyanidins, have an upregulation of the expression of genes associated with phenylpropanoid, stilbene and flavonoid pathways. Also, genes encoding to the machinery involving the accumulation of anthocyanins in the vacuole were also upregulated in transgenic cells (Figure 9). The proper regulation of these metabolic pathways is crucial to ensure better berry quality, that consequently is translated into better wine quality and production of better products by the winemaking industry.

Despite the importance of these findings, some challenges remain. For instance, regenerating grapevine plants from these non-embryogenic VviNAC17-overexpressing cells might prove to be a difficult task. This work would also have benefit from an assessment of a direct physical interaction of VviNAC17 with its target genes, for instance *via* binding assays such as EMSA (Irfan et al., 2014).

Indeed, the accumulation of these secondary metabolites and the underlying upregulation of their biosynthetic pathways *via* the action of VviNAC17 is very relevant from an agronomic and/or nutritional standpoint. Anthocyanin and proanthocyanidin concentrations, for instance, are very important quality traits of wines. Also, the future perspective of having transgenic grapevines overexpressing VviNAC17, in addition to a higher grape berry quality scenario, might represent an opportunity to cultivate plants more able to resist to biotic stress due to their significantly increased synthesis of stilbenes and other phenolics, metabolites known to have an important role against pathogen attacks (Latouche et al., 2013; Viret et al., 2018). Parallel to this important agronomical added value, the function of VviNAC17, *via* its overexpression, might be harnessed to develop and optimize an *in vitro* strategy to overproduce these secondary metabolites with important bioactive and nutraceutical properties using Gamay Fréaux grape berry cells, that can in the future lead to their sustainable production, something that would greatly benefit agrifood, nutraceuticals and even the cosmetics industry.

## Data availability statement

The original contributions presented in the study are included in the article/Supplementary material, further inquiries can be directed to the corresponding author.

## Author contributions

HB performed the experiments and wrote the manuscript. MV and MC performed the experiments. AG and HG advised, wrote, and reviewed the manuscript. AC conceptualized the work, performed the experiments, and wrote and reviewed the manuscript. All authors contributed to the article and approved the submitted version.

## Funding

This work was supported by Fundação para a Ciência e Tecnologia (FCT), under the strategic program UIDB/BIA/04050/2020. This work was also supported by FCT and European Funds (FEDER/POCI/COMPETE2020) through the research project “MitiVineDrought—Combining ‘omics’ with molecular, biochemical, and physiological analyses as an integrated effort to validate novel and easy-to-implement drought mitigation strategies in grapevine while reducing water use” with ref. PTDC/BIA-FBT/30341/2017 and ref. POCI-01-0145-FEDER-030341; AC was supported with a post-doctoral researcher contract/position within the project “MitiVineDrought” (ref. PTDC/BIA-FBT/30341/2017 and ref. POCI-01-0145-FEDER-030341). HB was supported by a Ph.D. fellowship funded by FCT (SFRH/BD/144638/2019). MV was supported by a Ph.D. fellowship funded by FCT (SFRH/BD/144637/2019).

## Conflict of interest

The authors declare that the research was conducted in the absence of any commercial or financial relationships that could be construed as a potential conflict of interest.

## Publisher’s note

All claims expressed in this article are solely those of the authors and do not necessarily represent those of their affiliated organizations, or those of the publisher, the editors and the reviewers. Any product that may be evaluated in this article, or claim that may be made by its manufacturer, is not guaranteed or endorsed by the publisher.

## Supplementary material

The Supplementary material for this article can be found online at: https://www.frontiersin.org/articles/10.3389/fpls.2022.964621/full#supplementary-material
