# Supplementary material for: Constitutive expression of VviNAC17 transcription factor significantly induces the synthesis of flavonoids and other phenolics in transgenic grape berry cells
Source: Front Plant Sci. 2022 Jul 29;13:964621. doi: 10.3389/fpls.2022.964621 (PMC9372392; doi:10.3389/fpls.2022.964621)
Supplement: Supplementary file 1 [file Data_Sheet_1.PDF]

Supplementary Material

Table 1. *VviNAC17* Gateway® cloning primers.

**For. Primer**	5'-GGGGACAAGTTTGTACAAAAAAGCAGGCTCGGAAATGGGTGTACCGGAGAC-3'
**Rev. Primer**	5'- GGGGACCACTTTGTACAAGAAAGCTGGGTTCTGCCTATATCCAAATCCACT-3'

Table 2. Real-time qPCRs primers.

Gene	Accession Number	Primers	Reference
VviPAL1	Vitvi08g01022	F: 5′-CCGAACCGAATCAAGGACTG-3′	Boubakri *et al.* (2013)
		R: 5′-GTTCCAGCCACTGAGACAAT-3′	
VviSTS1	Vitvi16g01486	F: 5′-CGAAGCAACTAGGCATGTGT-3′	Boubakri *et al.* (2013)
		R: 5′-CTCCCCAATCCAATCCTTCA-3′	
VviCHS1	Vitvi02g01015	F: 5′-GTCCCAGGGTTGATTTCCAA-3′	Boubakri *et al.* (2013)
		R: 5′-TCTCTTCCTTCAGACCCAGTT-3′	
VviFLS1	Vitvi18g02542	F: 5'-CAGGGCTTGCAGGTTTTTAG-3'	Downey *et al.* (2003)
		R: 5'-GGGTCTTCTCCTTGTTCACG-3'	
VviDFR	Vitvi18g00988	F: 5′-GGCTTTCTAGCGAGAGCGTA-3′	Bogs *et al.* (2006)
		R: 5′-ACTCTCATTTCCGGCACATT-3′	
VviLDOX	Vitvi13g00055	F: 5'-ACCTTCATCCTCCACAACAT – 3'	Bogs *et al.* (2005)
		R: 5'-AGTAGAGCCTCCTGGGTCTT – 3'	

VviLAR1	Vitvi01g00234	F: 5′-CAGGAGGCTAAGATAC-3′	Bogs *et al.* (2005)
		R: 5′-ACGCTTCTCTATGTTG-3′	
VviANR	Vitvi10g02185	F: 5′-CAATACCAGTCTGAGC-3′	Bogs *et al.* (2005)
		R: 5′-AAACTGAACCTTTCAC-3′	
VviUFGT1	Vitvi16g00156	F: 5’–TGCAGGGCCTAACTCACTCT–3	Designed with the aid of QuantiPrime (Arvidsson et al. (2008))
		R: 5’–GCAGTCGCCTTAGGTAGCAC–3	
VviGST4	Vitvi04g00880	F: 5’- AAGGATCCATGGTGATGAAGGTGTATGGC-3'	Conn *et al.* (2008)
		R: 5’- AACTGCAGAAGCCAACCAACCAACAAAC–3’	
VviMYBA1	Vitvi02g01019	F: 5′-AGAACAGGTTCGAGGTTG-3′	Xie *et al.* (2020)
		R: 5′-TCTATTCAACCCTGCTCGG-3′	
VviMYBA2	Vitvi02g01015	F: 5’-GCAGGGTTGAATAGATGCCTAAA-3’	Niu *et al.* (2016)
		R: 5’-CTCGTCTAATGCAAACTCTCCTCTC-3’	
VviMYBPA1	Vitvi13g00055	F: 5′-AGATCAACTGCTTGCT-3′	Bogs *et al.* (2007)
		R: 5′-AACACAAATGGCACAC-3′	
VviMYB14	Vitvi07g00598	5’-TCTGAGGCCGGATATCAAAC-3’	Höll *et al.* (2013)
		5’-GGGACGCATCAAGAGAGTGT-3’	
VviMYB15	Vitvi05g01733	5’-CAAGAATGAACAGATGGAGGAG-3’	Höll *et al.* (2013)
		5’-TCTGCGACTGCTGGGAAA-3’	
VviMATE1	Vitvi16g01911	F: 5’- TGCTTTTGTGATTTTGTTAGAGG-3’	Gomez *et al.* (2009)
		R: 5’-CCCTTCCCCGATTGAGAGTA-3’	

VviNAC17	Vitvi19g00270	F: 5′-GTCATCGTCGTCCCACCTC-3′	Ju *et al.* (2020)
		R: 5′-AAGAACCTGTCATCGATCTCC-3′	
VviACT1	Vitvi04g01613	F: 5’-GTGCCTGCCATGTATGTTGCC-3’	Conde *et al.* (2015)
		R: 5’-GCAAGGTCAAGACGAAGGATA-3’	
VviGAPDH	GSVIVG00009717001	F: 5’-CACGGTCAGTGGAAGCATCAT-3’	Conde *et al.* (2015)
		R: 5’-CCTTGTCAGTGAACACACCAG-3’	

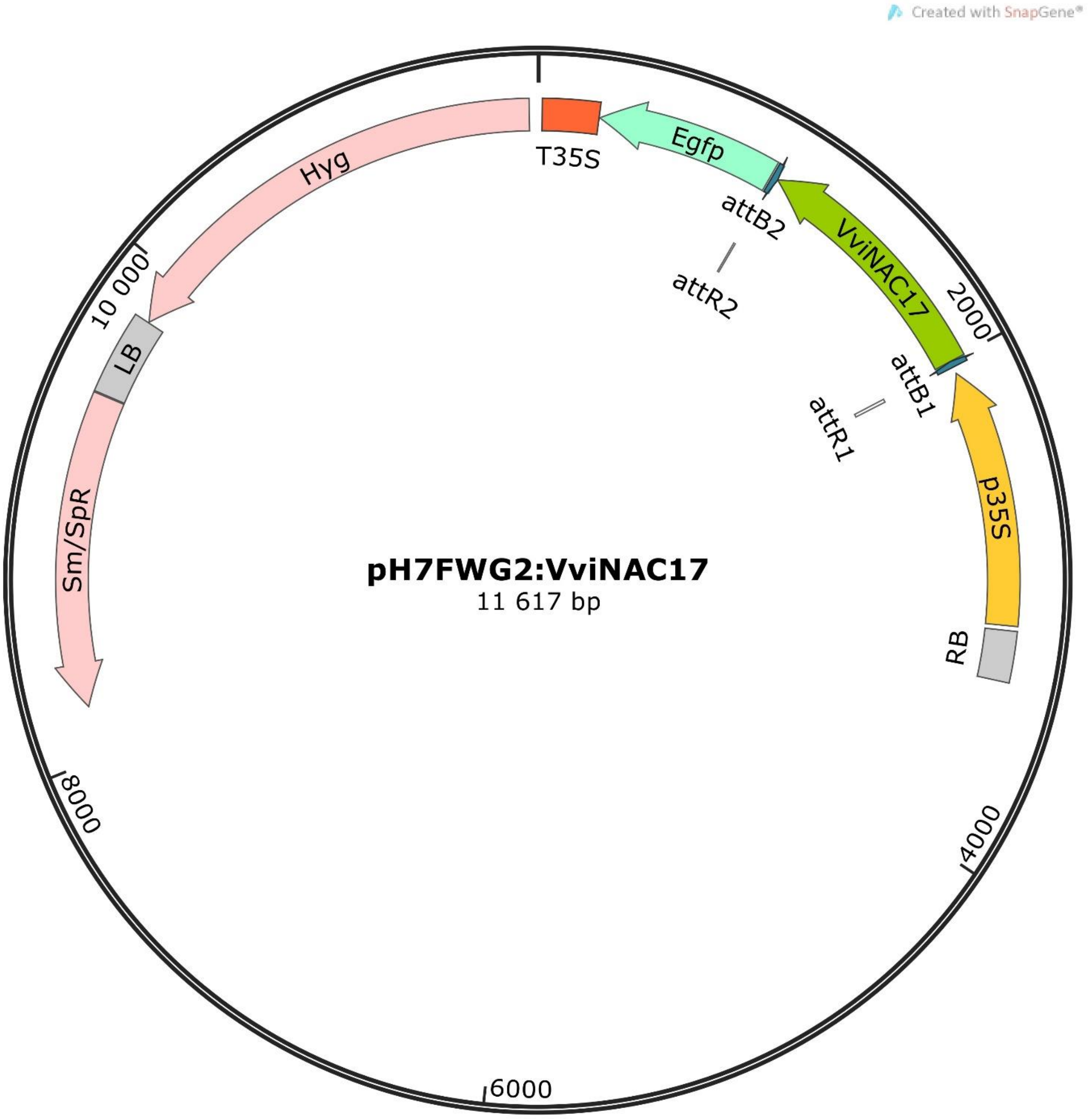

Figure 1 – T-DNA region of binary vector *pH7FWG2* (Karimi et al., 2002) containing *VviNAC17* used in Gamay transformation protocol. P35S, CaMV 35S promoter; **Hyg,** hygromycin phosphotransferase under the control of the pNOS promoter and tNOS terminator; **Egfp**, green fluorescent protein with the ER signal sequence p35S promoter; **T35S,** CaMV 35S terminator; **LB**, left border; **RB**, right border; **VviNAC17**, transcription factor NAC17 of *Vitis vinifera* introduced to eliminate ccdB gene from destination plasmid; **attB1** and **attB2**, attB sites of Gateway® recombination reactions.

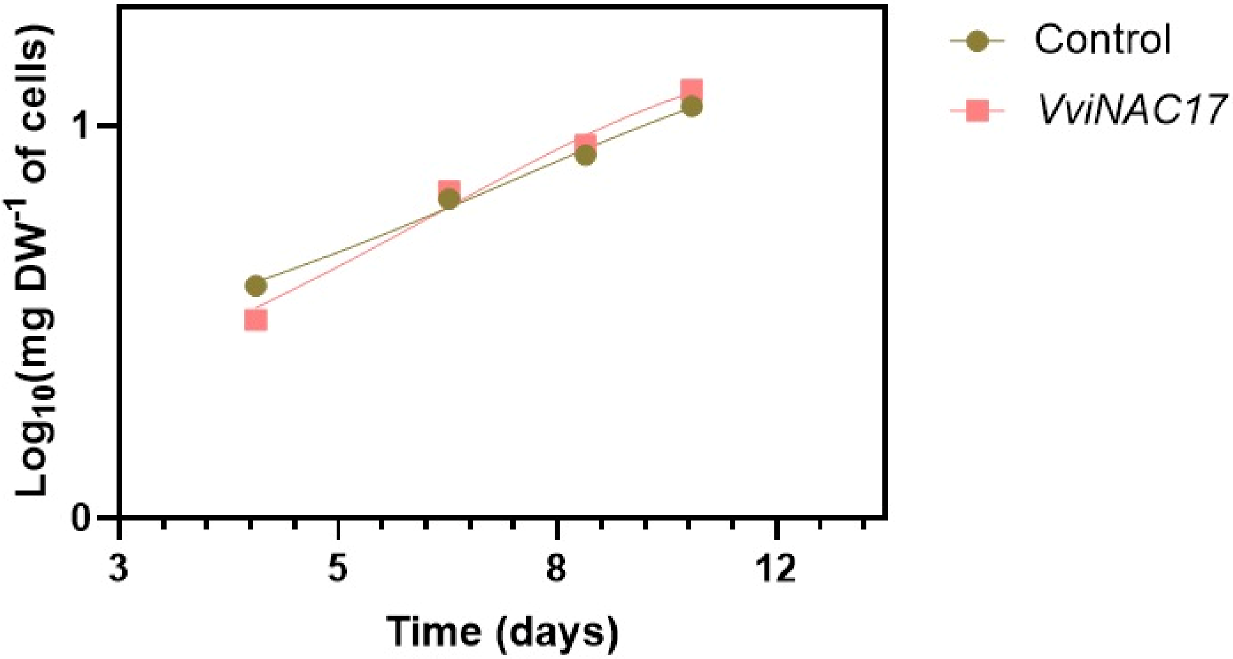

Figure 2 - Growth curve of Gamay cells and *VvNAC17*-overexpressing cells.

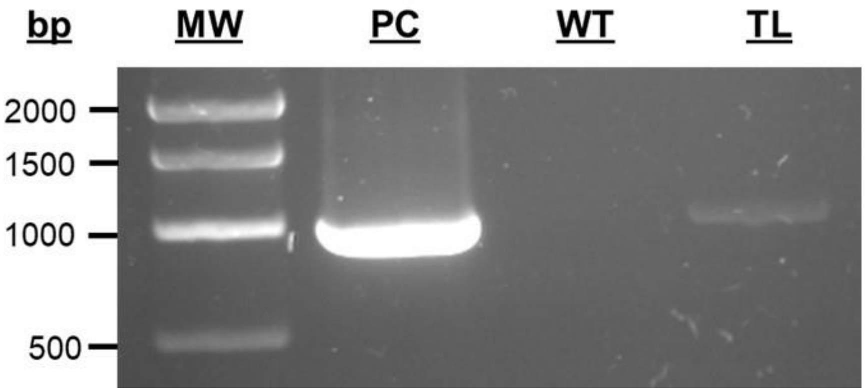

Figure 3 - PCR amplification products from genomic DNA of transgenic cell line of *Vitis vinifera* cv. Gamay. Amplification was carried out using p35S primers. **(PC)** Amplification products of positive control obtained by amplification of linearized plasmid DNA of *Agrobacterium.* **(WT)** Amplification products of negative control using a non-

transgenic genomic DNA (wild-type) of *Vitis* cells. **(TL)** Amplification products of p35S from suspension transgenic line of *Vitis vinifera* cv. Gamay.

REFERENCES

Bogs, J., Downey, M. O., Harvey, J. S., Ashton, A. R., Tanner, G. J., & Robinson, S. P. (2005). Proanthocyanidin Synthesis and Expression of Genes Encoding Leucoanthocyanidin Reductase and Anthocyanidin Reductase in Developing Grape Berries and Grapevine Leaves. *Plant Physiology, 139*(2), 652-663. doi:10.1104/pp.105.064238

Bogs, J., Ebadi, A., McDavid, D., & Robinson, S. P. (2006). Identification of the Flavonoid Hydroxylases from Grapevine and Their Regulation during Fruit Development. *Plant Physiology, 140*(1), 279-291. doi:10.1104/pp.105.073262

Bogs, J., Jaffé, F. W., Takos, A. M., Walker, A. R., & Robinson, S. P. (2007). The Grapevine Transcription Factor VvMYBPA1 Regulates Proanthocyanidin Synthesis during Fruit Development. *Plant Physiology, 143*(3), 1347-1361. doi:10.1104/pp.106.093203

Boubakri, H., Poutaraud, A., Wahab, M. A., Clayeux, C., Baltenweck-Guyot, R., Steyer, D., Marcic, C., Mliki, A., & Soustre-Gacougnolle, I. (2013). Thiamine modulates metabolism of the phenylpropanoid pathway leading to enhanced resistance to Plasmopara viticolain grapevine. *BMC Plant Biology, 13*(1), 31. doi:10.1186/1471-2229-13-31

Conde, A., Regalado, A., Rodrigues, D., Costa, J. M., Blumwald, E., Chaves, M. M., & Gerós, H. (2015). Polyols in grape berry: transport and metabolic adjustments as a physiological strategy for water-deficit stress tolerance in grapevine. *J Exp Bot, 66*(3), 889-906. doi:10.1093/jxb/eru446

Conn, S., Curtin, C., Bézier, A., Franco, C., & Zhang, W. (2008). Purification, molecular cloning, and characterization of glutathione S-transferases (GSTs) from pigmented Vitis vinifera L. cell suspension cultures as putative anthocyanin transport proteins. *J Exp Bot, 59*(13), 3621-3634. doi:10.1093/jxb/ern217

Downey, M. O., Harvey, J. S., & Robinson, S. P. (2003). Synthesis of flavonols and expression of flavonol synthase genes in the developing grape berries of Shiraz and Chardonnay (Vitis vinifera L.). *Australian Journal of Grape and Wine Research, 9*(2), 110-121. doi:https://doi.org/10.1111/j.1755-0238.2003.tb00261.x

Gomez, C., Terrier, N., Torregrosa, L., Vialet, S., Fournier-Level, A., Verriès, C., Souquet, J.-M., Mazauric, J.-P., Klein, M., Cheynier, V. r., & Ageorges, A. s. (2009). Grapevine MATE-Type Proteins Act as Vacuolar H+-Dependent Acylated Anthocyanin Transporters *Plant Physiology, 150*(1), 402-415. doi:10.1104/pp.109.135624

Höll, J., Vannozzi, A., Czemmel, S., D'Onofrio, C., Walker, A. R., Rausch, T., Lucchin, M., Boss, P. K., Dry, I. B., & Bogs, J. (2013). The R2R3-MYB Transcription Factors MYB14 and MYB15 Regulate Stilbene Biosynthesis in Vitis vinifera *The Plant cell, 25*(10), 4135-4149. doi:10.1105/tpc.113.117127

Ju, Y. L., Yue, X. F., Min, Z., Wang, X. H., Fang, Y. L., & Zhang, J. X. (2020). VvNAC17, a novel stress-responsive grapevine (Vitis vinifera L.) NAC transcription factor, increases sensitivity to abscisic acid and enhances salinity, freezing, and drought tolerance in transgenic Arabidopsis. *Plant Physiol Biochem, 146*, 98-111. doi:10.1016/j.plaphy.2019.11.002

Karimi, M., Inzé, D., & Depicker, A. (2002). GATEWAY™ vectors for Agrobacterium-mediated plant transformation. *Trends in Plant Science, 7*(5), 193-195. doi:https://doi.org/10.1016/S1360-1385(02)02251-3

Niu, T. Q., Gao, Z. D., Zhang, P. F., Zhang, X. J., Gao, M. Y., Ji, W., Fan, W. X., & Wen, P. F. (2016). MYBA2 gene involved in anthocyanin and flavonol biosynthesis pathways in grapevine. *Genet Mol Res, 15*(4). doi:10.4238/gmr15048922

Xie, S., Lei, Y., Chen, H., Li, J., Chen, H., & Zhang, Z. (2020). R2R3-MYB Transcription Factors Regulate Anthocyanin Biosynthesis in Grapevine Vegetative Tissues. *Front Plant Sci, 11*, 527.
